# Heterogeneous susceptibility of circulating SIV isolate capsids to HIV-interacting factors

**DOI:** 10.1186/1742-4690-10-77

**Published:** 2013-07-24

**Authors:** João I Mamede, Marc Sitbon, Jean-Luc Battini, Valérie Courgnaud

**Affiliations:** 1 Institut de Génétique Moléculaire de Montpellier UMR 5535 CNRS, 1919 route de Mende, 34293, Montpellier cedex 5, France; 2 Université Montpellier 2, Place Eugène Bataillon, 34095, Montpellier cedex 5, France; 3 Université Montpellier 1, 5 Bd Henry IV, 34967, Montpellier cedex 2, France

**Keywords:** HIV-1, HIV-2, SIV, TRIM5α, TRIMCyp, Nup153, Nup358/RanBP2, Lentiviral capsids

## Abstract

**Background:**

Many species of non-human primates in Africa are naturally infected by simian immunodeficiency viruses (SIV) and humans stand at the forefront of exposure to these viruses in Sub-Saharan Africa. Cross-species transmission and adaptation of SIV to humans have given rise to human immunodeficiency viruses (HIV-1 and HIV-2) on twelve accountable, independent occasions. However, the determinants contributing to a simian-to-human lasting transmission are not fully understood. Following entry, viral cores are released into the cytoplasm and become the principal target of host cellular factors. Here, we evaluated cellular factors likely to be involved in potential new SIV cross-species transmissions. We investigated the interactions of capsids from naturally circulating SIV isolates with both HIV-1 restricting (i.e. TRIM5 proteins) and facilitating (i.e. cyclophilin A and nucleopore-associated Nup358/RanBP2 and Nup153) factors in single-round infectivity assays that reproduce early stages of the viral life-cycle.

**Results:**

We show that human TRIM5α is unlikely to prevent cross-species transmission of any SIV we tested and observed that the SIV CA-CypA interaction is a widespread but not a universal feature. Moreover, entry in the nucleus of different SIV appeared to follow pathways that do not necessarily recruit Nup358/RanBP2 or Nup153, and this regardless of their interaction with CypA. Nevertheless, we found that, like HIV-1, human-adapted HIV-2 infection was dependent on Nup358/RanBP2 and Nup153 interactions for optimal infection. Furthermore, we found that, unlike HIV CA, SIV CA did not require a direct interaction with the Cyp-like domain of Nup358/RanBP2 to carry out successful infection.

**Conclusions:**

Circulating SIV present a variety of phenotypes with regard to CA-interacting restricting or facilitating factors. Altogether, we unveiled unidentified pathways for SIV CA, which could also be exploited by HIV in different cellular contexts, to drive entry into the nucleus. Our findings warrant a closer evaluation of other potential defenses against circulating SIV.

## Background

Retroviruses are known to overcome species barriers and there is now significant evidence that the AIDS epidemics resulted from several cross-species transmissions from primates to humans. Simian immunodeficiency viruses (SIV) form a large group of related viruses which are naturally endemic to a wide variety of African nonhuman primate species. To date, serological evidence of SIV infection has been reported in at least 45 Old World monkey and ape species and partial or full-length viral sequences have been characterized from most of them [1]. SIV have a long history of simian-to-simian transmissions [2-4]. Thus, the progenitor of the human immunodeficiency virus type 1 (HIV-1), SIVcpz, isolated from chimpanzees, is a recombinant virus derived from two viruses that infect Old World monkey species [5]. Moreover, at least 11 independent cross-species transmissions of SIV from chimpanzees (*Pan troglodytes troglodytes*) and sooty mangabeys (*Cercocebus atys*) to humans have given rise to HIV-1 and HIV-2, respectively [6-8]. More recently a twelfth independent transmission has been described with the isolation of a new group of HIV-1, P, in a Cameroonian woman. HIV-1 P is closely related to an SIV found in gorillas (*Gorilla gorilla*) in the wild [9]. Therefore, a large pool of SIV that are widely distributed in Sub-Saharan Africa represents a potential risk for the exposed human population [10,11]. However, despite this high exposure to viruses, successful SIV cross-species infection remains rather rare since the majority of the SIV characterized so far, have no known human counterparts. Nevertheless, little is known about the factors that can potentially contribute to, or restrict, simian-to-human transmissions. The SIV tropism for new species is determined by their ability to exploit particular cellular host proteins for their replication while counteracting inhibiting factors. In primates, several classes of retroviral cellular restrictions have been identified at different steps of the retroviral cycle, including cytidine deaminases (*e*.*g*. APOBEC3G) [12], CD317/tetherin [13,14], SAMHD1 [15,16] and TRIM5α proteins [17]. Interestingly, it has been shown that all these proteins display signatures of positive selection throughout many primate lineages [18-21] indicative of an ongoing host/virus history.

Following binding of virions to cellular receptors and entry into the cell, the viral capsid (CA) is released into the cytoplasm. Thus, CA constitutes an early target for host cellular factors that are either required for lentivirus replication or have anti-retroviral properties. One of the earliest identified post-entry restriction factors, TRIM5α, acts before reverse transcription [17]. TRIM5α is a member of the large family of tripartite motif proteins (TRIM) that contain RING, B-box, and coiled-coil domains with an additional C-terminal PRYSPRY domain, which is required for its antiretroviral activity [22]. TRIM5α restricts retroviruses in a virus-specific and species-specific way. For example, rhesus monkey (rh) or African green monkey (agm) TRIM5α potently restricts HIV-1, whereas human TRIM5α has no significant detectable effect on HIV-1; in contrast human TRIM5α potently restricts N-tropic murine leukemia virus (N-MLV) [23-25], a widespread and phylogenetically very distant mouse gammaretrovirus. Interestingly, it has recently been described that some HIV-2 strains are sensitive to human TRIM5α [26]. TRIM5α is a cellular E3 ubiquitin ligase, that recognizes incoming multimerized viral cores and accelerates uncoating of retroviral capsids [27,28]. More recently, it has been described that TRIM5α can act as a pattern recognition receptor (PRR), promoting innate immune signaling in response to retroviral infection [29]. The CA protein of HIV-1 also interacts with high affinity to the host protein cyclophilin A (CypA) [30]. CypA, encoded by the peptidyl prolyl isomerase A gene (PPIA), has been shown to be important for efficient HIV-1 replication either by promoting uncoating [31] or by protecting the CA from an unidentified restriction factor [32-34]. Intriguingly, in the New World owl monkeys (*Aotus sp*.), TRIM5α is replaced by TRIMCyp in which a CypA pseudogene is fused to the Linker 2 region, substituting for the SPRY domain [35,36]. More recently, similar but independent TRIM-Cyp gene fusions have been also discovered in different species of Old World monkeys, such as rhesus macaques (*Macaca mulatta*) and cynomolgus macaques (*Macaca fascicularis*), highlighting the reproducible evolutionary advantages provided by this gene fusion [37]. Owl-TRIMCypA and mafa-TRIMCypA proteins were described to efficiently block HIV-1 infection while mamu-TRIMCypA was shown to block infection by HIV-2 [38]. Therefore, CypA binding to viral CA is an evolutionary widespread feature and likely to be required by multiple HIV/SIV lineages.

Recently, large-scale RNA interference screens have revealed numerous host protein factors influencing HIV-1 infection, bringing to light potential new pathways in the viral life cycle [39-43]. In contrast to other retroviruses, lentiviruses have developed specific mechanisms for the productive infection of non-dividing cells [44] and it has been shown that the capsid plays an important role for this property of HIV-1 [45-48]. Alhough the mechanisms used by the HIV-1 pre-integration complexes (PIC) to enter into the nucleus are not fully understood, a small overlap between independent screens highlight several critical co-factors known to play independently a role in the nuclear import of the HIV-1 PIC [49-51]. Indeed, depletion of the nuclear pore complex component Nup153 or the nuclear transport factor Nup358/RanBP2 hindered nuclear import of HIV-1 and SIVmac [52-55]. Additionaly, it has been shown that depletion of the karyopherin transportin 3 (TNPO3) strongly decreases the infectivity of HIV-1 through a specific block of integration following nuclear import [53,56-58]. Nup153 and Nup358/RanBP2 are present in nuclear pore complexes (NPC) and seem to actively regulate nuclear entry of viral PIC [45,54,57,59,60]. Nup358/RanBP2, is located at the cytoplasmic side of the pore and has a C-terminal cyclophilin-like domain [61]. Given that HIV-1 can interact directly with this domain, it has been hypothesized that this motif could be responsible for the recognition of incoming capsids and thus mediating nuclear import [62,63].

Most of the knowledge regarding successive steps in lentiviral replication comes from studies using prototypic HIV-1 or SIVmac. However, much less is known on the intrinsic capabilities of SIV isolates that circulate in the wild to achieve productive infections in human cells, notably because corresponding infectious molecular clones are not available. Capsids play an essential role in the early stages of viral replication and, as highlighted in a multiple alignment of primate lentiviral CA protein sequences, they harbor both highly divergent and conserved residues, including within the CypA-binding loop.

Here, we have defined the susceptibilities of SIV CA to human or simian proteins known to interact with HIV-1 CA, i.e. the cytoplasmic TRIM5 proteins, CypA and the nucleopore-associated Nup358/RanBP2 and Nup153 factors. We chose to study SIV isolates amplified from small primates that are commonly hunted and sold as bushmeat in West Central Africa (SIVgsn lineage, SIVcol and SIVmnd1).

## Results

**Figure 1:**
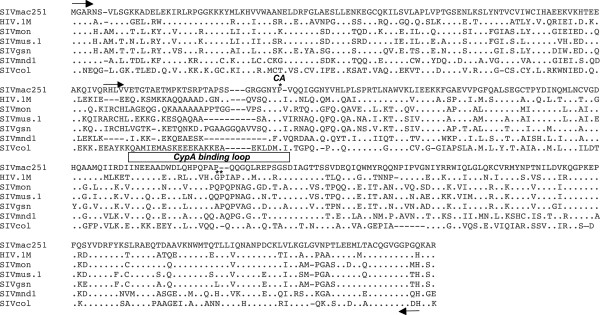
**Capsid sequences of circulating SIV are highly variable.** Alignment of matrix and capsid amino acid sequences of prototypic SIVmac251 and HIV-1 (pNL4-3) with those of SIV circulating in the wild, SIVmon-99CMCML1 accession No. AY340701 isolated from mona monkey (*Cercopithecus mona*); SIVgsn-99CM71 accession No. AF468658, from greater spot-nosed monkey (*Cercopithecus nictitans*); SIVmus-01CM1085 accession No. AY340700, from mustached monkey (*Cercopithecus cephus*), SIVmnd1, accession No. M27470, from mandrill (*Mandrillus sphynx*) and SIVcolCGU1 accession No. AF301156) from mantled guereza (*Colobus guereza*). Dots represent fully conserved residues and deletions are indicated with dashes. Arrows indicate the positions of the primers used to generate the chimeric GagPol constructs (MACA and CA). The beginning of CA is indicated by a star on top of the proline residue as well as the G89 and P90 residues; the CypA-binding loop is indicated on top of the corresponding residues.

**Figure 2:**
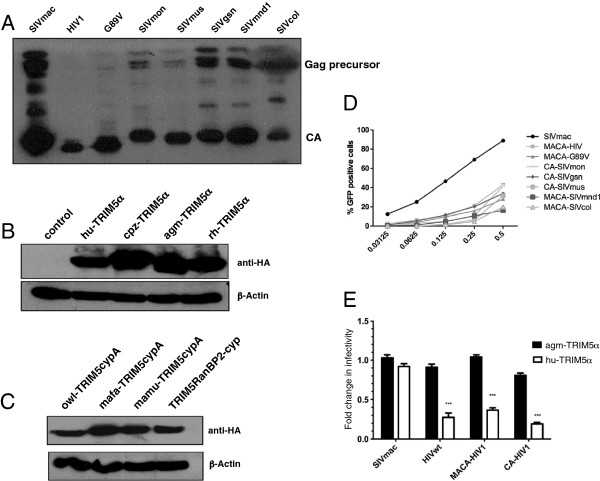
**Chimeric constructs that express lentiviral capsids and stable expression of primate TRIM5 in CHO cells reproduce TRIM5α restriction specificity. (A)** Wild-type SIVmac *GagPol* expression vector and chimeric *GagPol* constructions were used to obtain single-round infectious virions, containing an SIVmac vector with eGFP and the vesicular stomatitis virus G fusion protein. Supernatants were harvested 48 h later and pelleted by ultracentrifugation through a 20% sucrose cushion. Viral preparations that gave 20% GFP positive cells on CHO cells were immunoblotted. Gag proteins were detected using sera from HIV-2 infected patients. **(B)** Stable expression of TRIM5α proteins in CHO cells. Cell extracts were obtained from CHO cells and CHO cells transduced with vectors encoding HA-tagged TRIM5α from human (hu-TRIM5α), chimpanzee (cpz-TRIM5α), African green monkey (agm-TRIM5α), or rhesus monkey (rh-TRIM5α). Immunoblotting was performed with an anti-HA monoclonal antibody. Actin was probed as loading control. **(C)** Cell extracts were obtained from CHO cells transduced with vectors encoding HA-tagged TRIM5CypA proteins from owl monkey (owl-TRIMCypA), *Macaca mulatta* (mamu-TRIMCypA) or *Macaca fascicularis* (mamu-TRIMCypA). TRIM-RanBP2Cyp corresponds to a synthetic protein constructed after fusing TRIM5 Linker 2 region of owl-TRIMcypA with the human RanBP2-Cyp sequence. **(D)** Infection efficiency of serial dilutions for each chimera in CHO hamster cells. **(E)** Restriction specificity in TRIM5α-expressing CHO cell lines. CHO cells stably expressing hu-TRIM5α (solid bars) or agm-TRIM5α (open bars) were challenged with virions harboring wild-type SIVmac or HIV-1 CA, or chimeric SIVmac-HIV-1 (MACA or CA) Gag proteins. GFP-positive cells were enumerated by flow cytometry. Ratios of percentage of infected cells in TRIM5α-expressing CHO cells over CHO control cells were determined. Results are expressed as means from at least three independent experiments. Error bars represent the standard error of the mean. Unpaired two-tailed Student’s t-test was used to assess significance and P-values < 0.001) are indicated with asterisks.

In order to study the susceptibility of different SIV cores to human or simian TRIM5α, we derived *gag*-*pol* constructs with the SIVmac background in which we replaced CA with that of either HIV-1 or different circulating SIV. Because Gag protein sequences of lentiviral primate lineages are highly diverse (Figure 1), we first validated our strategy by inserting HIV-1 matrix-capsid (MACA) or capsid (CA) sequences in place of the corresponding region of an SIVmac251 *gag*-*pol* expression vector. Upon transfection into HEK-293T cells, VSV-G pseudotyped eGFP-expressing chimeric HIV MACA or CA virions were produced and we verified the correct p55Gag proteolytic cleavage by detecting the presence of CAp24 in virus pellets (Figure 2A). The infectivity of the corresponding virions was then tested on non-restrictive CHO cells and CHO cells stably transduced with retroviral vectors carrying either the human or one of the simian HA-tagged-TRIM5 genes. Similar amounts of TRIM5α (Figure 2B) and TRIM-Cyp (Figure 2C) proteins were expressed in the transduced cells. As shown in Figure 2E, the parental SIVmac vector infected equally well both CHO derivatives whereas infection by SIVmac constructs, in which HIV-1 MACA or CA were swapped, was decreased in agm-TRIM5α expressing cells, thus reproducing the restriction pattern observed with wild type HIV-1 CA. We subsequently substituted the MACA or CA-coding sequences of SIVmac by those of the different SIV of the SIVgsn lineage (SIVgsn from greater spot-nosed monkeys, SIVmon from mona monkeys and SIVmus-1 from mustached monkeys), SIVcol from Colobus guereza monkeys, SIVmnd1 from mandrills and a HIV-1 CypA binding mutant (CA G89V). All VSV-G pseudotyped eGFP-expressing chimeric MACA or CA SIV vectors were infectious on CHO cells and were subsequently used at M.O.I ranging from 0.05 to 0.35. For the ensuing studies, we chose the MACA or CA chimera that gave the highest titer for each of the SIV isolates studied (Figure 2D).

### CA from circulating SIV isolates are not susceptible to human or chimpanzee TRIM5α

**Figure 3:**
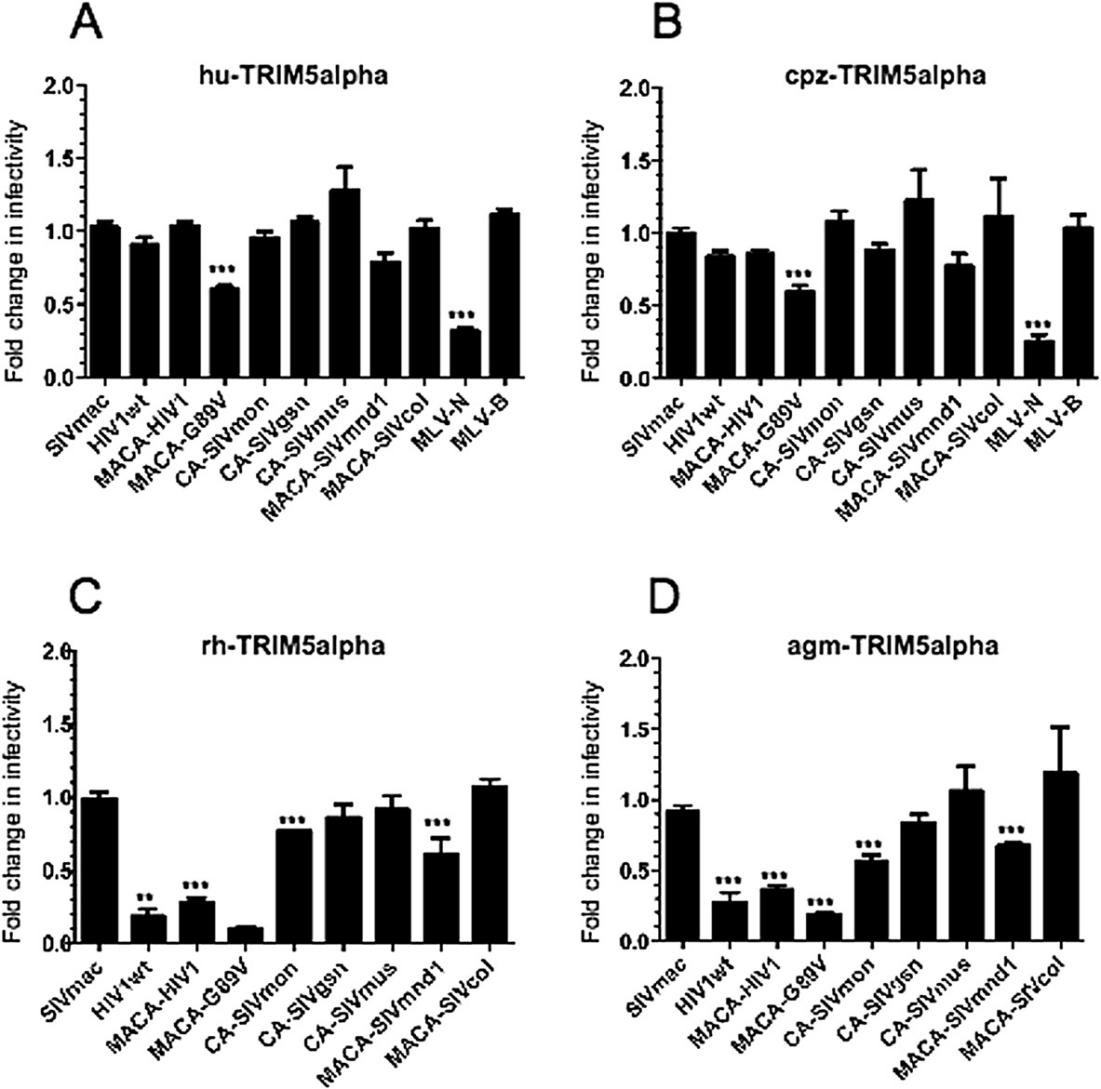
**Distinctive patterns of restriction are due to the types of primate TRIM5α isoform and the type of circulating SIV capsid that is targeted. (A)** Restriction profiles of different primate TRIM5α isoforms against circulating SIV CA are determined in control CHO cells or CHO cells stably expressing **(A)** the human, **(B)** the chimpanzee, **(C)** the rhesus, or **(D)** the African green monkey TRIM5α proteins. Virion preparation and infection efficiency were as described in the legend of Figure 2. Additional control virions were used, including those with the CA of TRIM5α-susceptible N-tropic MLV (MLV-N) and TRIM5α-resistant MLV-B. Results were obtained from at least three independent experiments for each combination. Unpaired two-tailed Student’s t-test was used to assess significance. SEM and P-values are indicated (** P < 0.01; *** P < 0.001).

Since amino acid sequence variations in CA determine viral susceptibility to TRIM5α restriction, we tested the inhibition pattern of human or simian TRIM5α proteins with circulating SIV isolates. We carried out single-round VSV-G pseudotyped eGFP virus infections of CHO cells stably expressing various TRIM5α alleles. Infectivity of a control N-tropic MLV, which is restricted by hu or cpz-TRIM5α, was significantly reduced (ratio 0.3, p < 0.001) while the B-tropic MLV negative control remained unaffected, thus confirming the restriction specificity of our model (Figure 3A and B). As shown in Figure 3A and B, the overall restriction profiles of hu and cpz-TRIM5α were very similar. With the exception of a partial restriction activity against HIV-G89V (ratio dropped to 0.6, p < 0.001), which has already been observed by others [32,64] none of the other primate lentiviruses tested were significantly restricted.

We next assessed the restriction patterns of the rh and agm-TRIM5α (Figure 3C and D). While SIVmac remained unrestricted, as expected, both rh and agm-TRIM5α significantly restricted all of the HIV-1 CA recombinants, with infectivity ratio dropping from 0.1 to 0.4 as compared to SIVmac (p < 0.01 and 0.001). Although the two primate TRIM5α alleles strongly restricted HIV-1 and HIV-G89V, restriction of the SIV isolates varied considerably. Thus, SIVmon and SIVmnd1 were significantly but only partially restricted (ratio of 0.6 or higher, p < 0.001) and SIVgsn, SIVmus-1 and SIVcol were not detectably restricted by either alleles of TRIM5α (Figure 3C and D). Remarkably, the pattern of susceptibility to TRIM5α of the different SIV did not parallel the virus phylogeny, as illustrated, for instance, by SIVmon that is closely related to SIVgsn and SIVmus-1.

### SIV CA-CypA interaction is a widespread but not a universal phenotype

**Figure 4:**
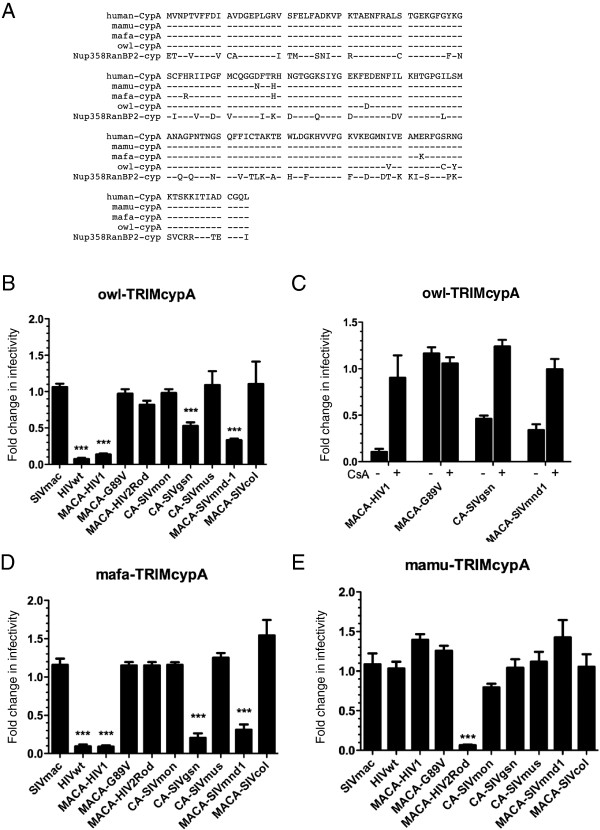
**Natural primate TRIMCypA fusion proteins show heterogeneous patterns of restriction against circulating SIV CA. (A)** Highly conserved sequences between human CypA and the carboxy terminal CypA domains of primate TRIM5CypA fusion proteins; also shown is the related, but more divergent, carboxy terminal Cyp-like domain of the Nup158/RanBP2 nucleoporin (*see text*); (−) identical residues. **(B)** Restriction pattern of a CHO cell line stably expressing owl-TRIMCypA fusion protein against different lentiviral capsids as compared to infection of control CHO cells. **(C)** assessment of CypA dependence of the owl-TRIMCypA restriction against prototypic wt and mutant HIV-1 capsid constructs and against two circulating SIV capsids, assayed in the absence (−) or the presence (+) of cyclosporine A (CsA), a CypA inhibitor. **(D)** Infection titers of chimeric CA viruses on CHO cells stably expressing either TRIMCypA from *Maccaca fascicularis* (mafa-TRIMCypA) or **(E)** TRIMCypA from *Maccaca mulatta* (mamu-TRIMCypA) were compared to titers on control CHO cells as described above in **(B)** with the additional use of a chimeric SIVmac *GagPol* construct containing MACA of HIV2_ROD_ as control. Infectivity was calculated as indicated in Figure 2. All results were compiled from at least three independent experiments for each combination. Unpaired two-tailed Student’s t-test was used to assess significance. SEM and P-values < 0.001 (asterisks) are indicated.

We next tested whether susceptibility of circulating SIV isolates to certain simian TRIM5α proteins correlated with CypA-CA interactions. CypA is a host cellular factor that is essential for efficient HIV-1 replication in human cells through its interaction with CA [30]. Several lentiviruses bind CypA with high affinity but others, such as SIVmac, do not [65,66]. We therefore investigated the ability of CA from circulating SIV isolates to interact with CypA by taking advantage of a TRIMCyp fusion protein naturally present in owl monkeys [35,36]. Owl-TRIMCyp harbors a CypA domain that shares 97% identity with human CypA (Figure 4A). Our model was further validated with the CHO cells that stably express owl-TRIMCyp as these cells reproduced the expected restriction of HIV-1 with no restriction against the HIV-1 G89V mutant, which does not bind CypA (Figure 4B). With regard to other circulating SIV, we found that infectivity of SIVmnd1 was considerably reduced (drop of infectivity ratio to less than 0.4, p < 0.001) and confirmed that SIVgsn was susceptible to owl-TRIMCyp restriction [67] (Figure 4B).

**Figure 5:**
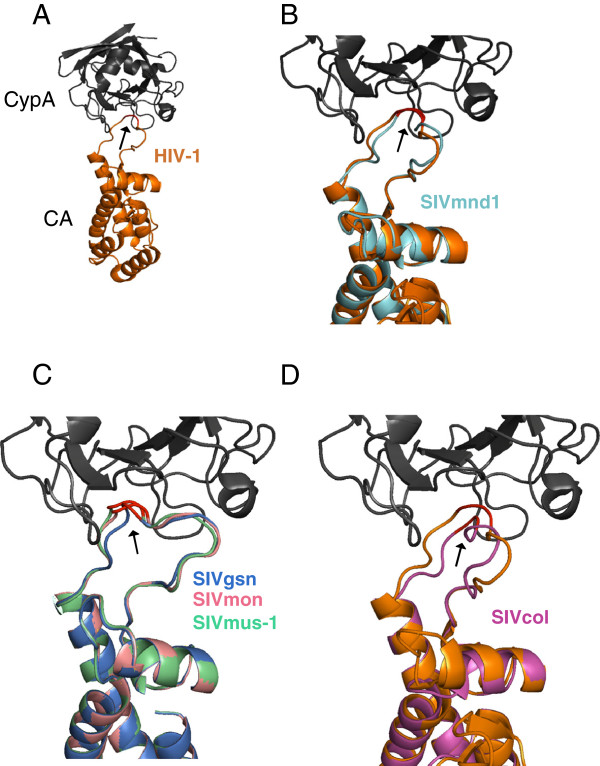
**Structure of lentiviral capsids bound to human CypA reveals distinctive groups of interactions amongst the different circulating SIV capsids. (A)** HIV-1 CA crystal structure (gold ribbon) bound to human CypA (grey ribbon) as compiled in the databanks (PDB: 1M9C; http://www.resbiorg/pdb/explore.do?structureId=1m9c). The Glycine and proline residues at position 89 and 90, respectively, are indicated in red and with an arrow on HIV-1 CA and at the equivalent positions on the different circulating SIV. HIV-1 CA-CypA crystal structure is used as template to predict the CA structure of different circulating SIV (see Figure 1 legend). **(B)** CA of SIVmnd-1, shown as a blue ribbon, is superimposed on HIV CA. **(C)** Superimposition of the three CA that belong to the SIVgsn lineage, SIVgsn, in blue; SIVmon, in pink; and SIVmus-1, in green. **(D)** CA of SIVcol, in pink, superimposed with HIV-1 CA.

We further verified that the TRIMCyp-mediated restriction in hamster cells was indeed due to binding to the CypA domain by using cyclosporine A (CsA), a competitive inhibitor which binds to CypA (Figure 4C). However, we found that the ability of SIVmnd1 and SIVgsn to infect human cells does not seem to involve a CypA dependence, since, in contrast to HIV-1, CsA treatment did not inhibit, but rather enhanced, infectivity (data not shown), in agreement with results obtained by others with SIVmac [68]. Amongst the SIV that we observed to be resistant to TRIMCyp restriction, were SIVcol and, interestingly, SIVmon and SIVmus-1 which are closely related to SIVgsn (Figure 4B). The CypA binding loop of lentiviral CA is the proline-rich sequence comprised between helices 4 and 5 on HIV-1 CA [69]. By using the CA crystal structure of HIV-1 bound to human CypA (PDB: 1M9C) (Figure 5A) and the raptorX server [70], we superimposed the predicted structure of the CA from SIVmnd1 (Figure 5B), those of the three SIV of the SIVgsn lineage (Figure 5C) and that of SIVcol (Figure 5D). We found that the CypA-binding loop of SIVmnd1 superimposed closely with that of HIV-1 while the SIVcol loop, which is shorter, does not (Figure 5B and D). Interestingly, as shown in Figure 5C, within the SIVgsn lineage, the change of A (CA SIVgsn) to Q (CA SIVmon and SIVmus-1) introduced a subtle conformational change, that significantly altered the direct contact with the hydrophobic pocket of CypA, even if the target proline ′90′ remained present in the middle of the loop. Therefore, structural predictions of CA-CypA interactions as shown in Figure 5 are in accordance with the TRIM-CypA restriction phenotype.

Curiously, while TRIMCyp has been first described in owl monkeys, a New-World primate, fusion of the CypA coding sequence with *TRIM5* has since been described in at least two Old-World macaques species (*mafa*-*TRIMCypA* in *Macaca fascicularis* and *mamu*-*TRIMCypA* in *Macaca mulatta*,), illustrating the strong recurrent selection pressure of this event during primate evolution [37]. As these different CypA sequences show some amino acid divergence (Figure 4A), we tested whether SIVgsn and SIVmnd1 were susceptible to mafa-TRIMCyp, like HIV-1, or rather, to mamu-TRIMCyp, like HIV-2. Interestingly, both SIVmnd1 and SIVgsn displayed an HIV-1-like phenotype, with a strong restriction measured with mafa-TRIMCyp (infectivity ratio below 0.4, p < 0.001), while neither was susceptible to mamu-TRIMCyp (Figure 4D and E). The specific restriction pattern of mamu-TRIMCyp was fully reproduced in our model since a very strong restrictive effect was observed, as expected, on the HIV-2 construct (infectivity ratio below 0.2, p < 0.001) (Figure 4E).

### Nup358/RanBP2 depletion decreases SIVmac, SIVgsn and SIVmnd1 infectivity

In a global siRNA screening study, Nup153 and Nup358/RanBP2, two nucleoporins of the nuclear pore complex (NPC) were identified as essential for nuclear import of HIV-1 DNA [42]. Interestingly, Nup358/RanBP2, which localizes at the cytoplasmic side of the NPC, possesses a CypA-like domain that has been suggested to play an important role in viral infectivity through direct binding to HIV-1 CA [63].

**Figure 6:**
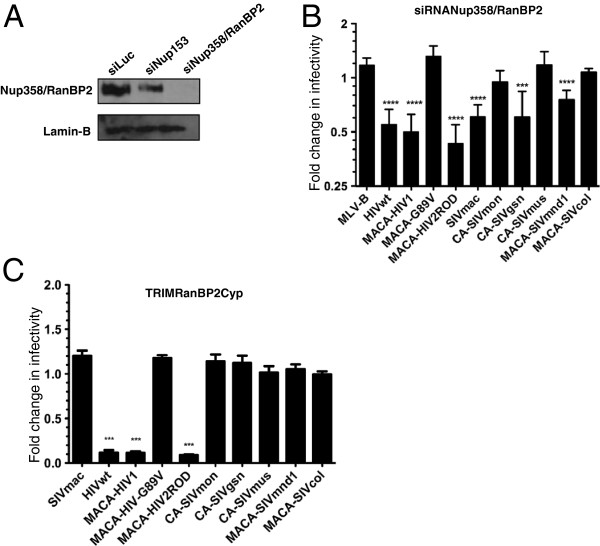
**HIV and circulating SIV capsids vary in their dependence on Nup358/RanBP2 for the optimal infection of human cells. (A)** Specific down modulation of Nup358/RanBP2 in HEK-293T cells by targeted siRNA. Whole-cell extracts of HEK-293T cells transfected with siRNA directed against either the luciferase gene (siLuc) or Nup153 (siNup153) or Nup358/RanBP2 (siNup358/RanBP2), were immunoblotted with a rabbit polyclonal antibody directed against Nup358/RanBP2 (upper panel) or a monoclonal antibody directed against lamin B, used as loading control (lower panel). **(B)** Infection of Nup358/RanBP2-depleted HEK-293T cells as compared to infection of siLuc-treated cells were determined as ratio of eGFP-positive cells as described in Figure 2 and Figure 3 legends using MLV-B, wt and chimeric HIV-1 constructs, HIV-2_ROD_, SIVmac, and circulating SIV capsid constructs. **(C)** Restriction activity in CHO cells stably expressing a synthetic TRIM-RanBP2Cyp fusion protein as compared to CHO cells. Cells were infected as described above with virions harboring CA from wt SIVmac and HIV-1, chimeric HIV-1 constructs, HIV-2_ROD_, and circulating SIV. All results were obtained from at least three independent experiments with each CA. Unpaired two-tailed Student’s t-test was used to assess significance. SEM and P-values < 0.001 (asterisks) are indicated.

In the context of our study, we examined whether human Nup358/RanBP2 interacted with the CA of the circulating SIV and investigated the impact of Nup358/RanBP2 depletion on infection with our chimeric CA viruses. Nup358/RanBP2 siRNA were transfected into HEK-293T cells and the knockdown of the protein was verified by immunoblotting using a Nup358/RanBP2 specific antibody (Figure 6A). Consistent with previous reports, HIV-1 was affected by Nup358/RanBP2 depletion while, on the contrary, infectivity of HIV-1 G89V slightly increased (Figure 6B). Furthermore, we observed that infectivity of SIVgsn and SIVmnd1 were also significantly reduced (p < 0.001) after Nup358/RanBP2 depletion. In contrast, SIVmus-1, SIVmon and SIVcol infections remained unaffected. Nevertheless, despite this pattern, there was no strict relationship between dependence on Nup358/RanBP2 for optimal infection and CypA-CA interaction. Indeed, in contrast with previous findings that used an shRNA approach [63], we found that owl-TRIMCyp insensitive SIVmac251 was also significantly susceptible to Nup358/RanBP2 depletion (Figure 6B). Moreover, this was also the case for HIV-2_ROD,_ which was sensitive to Nup358/RanBP2 depletion.

We further assessed whether susceptibility to Nup358/RanBP2 depletion in HEK-293T cells was related to binding to the CypA-like domain of the protein. For this purpose, we generated a synthetic TRIM-RanBP2Cyp protein that comprised the RBCC domain of owl-TRIMCypA in which we replaced the original owl CypA domain by that of Nup358/RanBP2 (Figure 4A). Stable expression of this TRIM-Nup358/RanBP2 CypA fusion protein in CHO cells was confirmed by western blot using an anti-HA antibody (Figure 2C). In agreement with a previous report, we observed that HIV-1 was tightly restricted by TRIM-RanBP2Cyp [63]. However, we found that, with the exception of HIV-2_ROD_, none of HIV-1 G89V, SIVmac or the circulating SIV isolates were restricted by TRIM-RanBP2Cyp (Figure 6C). These results indicate that the direct interaction of Nup358/RanBP2 by circulating SIV does not appear to be mediated via its Cyp-like domain. Whether the Cyp-like domain of Nup358/RanBP2 is actually required for HIV-1 infectivity in vivo is still subject to debate [62,71].

### Circulating SIV do not necessarily use Nup153 or Nup358/RanBP2, in contrast to both HIV types

**Figure 7:**
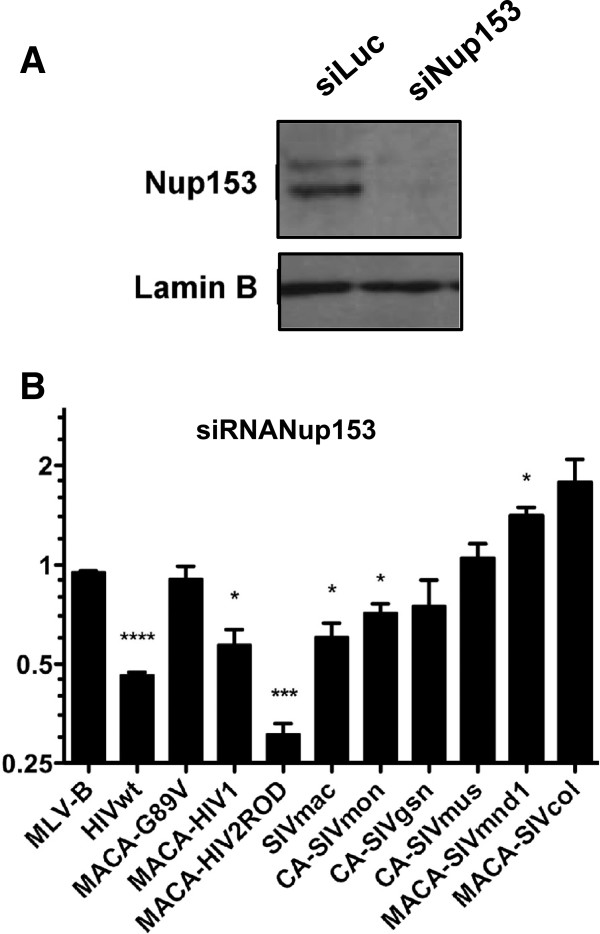
**Optimal infection of human cells mediated by both HIV-1 and HIV-2 capsids is dependent on Nup153 while capsids of SIVmac and circulating SIV capsids are mildly or not dependent on Nup153. (A)** Specific down modulation of Nup153 in HEK-293T cells (siRNANup153) as compared to control siLuc-treated cells. Cells were analyzed as described in the Figure 6 legend. **(B)** Infection of Nup153-depleted HEK-293T cells as compared to infection of control siLuc-treated cells were determined as described above using MLV-B, wt and chimeric HIV-1 constructs, HIV-2_ROD_, SIVmac, and circulating SIV CA constructs. Results were obtained from at least three independent experiments with each CA. Unpaired two-tailed Student’s t-test was used to assess significance. SEM and P-values (* P < 0.05, *** P < 0.001) are indicated.

Nup358/RanBP2 and Nup153 are on opposing sides of the NPC, respectively cytoplasmic or nuclear side [72,73]. Depletion of Nup153 inhibits HIV-1 infection and CA is involved in this dependence [54]. CypA appears to also play a role in directing HIV-1 into a nuclear entry pathway that involves Nup358/RanBP2 and in which recruitment of Nup153 plays a role [52,63]. Since our results on Nup358/RanBP2 dependence of viruses harboring Gag domains derived from different SIV isolates do not follow this observation, we further tested the infectivity of the Nup358/RanBP2-susceptible SIVmac, SIVgsn, SIVmnd1 on Nup153-depleted cells. In parallel, we monitored the infectivity of the Nup358/RanBP2-insensitive SIVmon, SIVmus-1, and SIVcol. Protein expression level was checked by immunoblotting using a Nup153 specific antibody (Figure 7A). In addition, we verified that Nup153 depletion did not impact on the expression level of Nup358/RanBP2 by immunoblotting using a Nup358/RanBP2 specific antibody (Figure 6A). In agreement with others’ results, we found that both HIV-1 and HIV-2_ROD_ infection of Nup153 knockdown HEK-293T cells was significantly inhibited while HIV-1 G89V infection remained unaffected as compared to the infection of control cells (Figure 7B). Moreover, Nup153 depletion slightly inhibited infection by SIVmac (ratio 0.5; p < 0.05) and SIVmon (ratio 0.7; p < 0.05), with either no significant inhibitory effect on the other circulating SIV or even a significantly increased infection as observed for SIVmnd1 (ratio 1.5; p < 0.05) (Figure 7B). This opposite effect on SIVmnd1 infection as compared to the Nup358/RanBP2 knockdown suggested that Nup153 may interfere with the engagement of another nucleoporin preferentially recruited by some of the circulating SIV.

Overall, CA appear to dictate the use of alternative pathways that can substitute for the CypA, Nup358/RanBP2 and Nup153 chain of engagement in the nuclear import of lentiviruses in human cells. However, dependence on the CypA, Nup358/RanBP2 and Nup153 CypA pathway seems to be conserved in both HIV-1 and HIV-2 although they are of different zoonotic origins. Taken ltogether, our results question whether this pathway reflects a required adaptation to human cells for efficient lentiviral infection.

## Discussion

Despite high exposure to lentiviruses of human populations in Sub-Saharan Africa, zoonotic transmissions of SIV do not appear to be so common [74]. Here, we evaluated the likelihood of new cross-species transmissions by investigating the interactions of CA from naturally circulating SIV isolates to cellular host factors that block or enhance HIV-1 replication at the early stages of the viral life-cycle. We found that TRIM5α, one of the species-specific barriers to HIV-1 in monkeys, is unlikely to provide a protection from the crossing of circulating primate lentiviruses to humans. So far, no SIV is known to be restricted by hu-TRIM5α and none of the CA from circulating SIV isolates tested here, including SIVcol, the most divergent SIV, were susceptible to either human or chimpanzee TRIM5α. This was not surprising given that the PRYSPRY domain of cpz-TRIM5α is closely related to that of hu-TRIM5α. SIVcpz, the ancestor of the HIV-1 groups M and N, emerged from the recombination of two lineages of SIV that infect monkeys [5,75]. The CA-containing 5′ part of the SIVcpz genome is related to SIV found in red-capped mangabey [76] whereas the env gene-containing 3′ part is related to the SIVgsn lineage, a lineage that comprises SIV that infect three *Cercopithecus* species [5,77]. Therefore, since the CA of the phylogenetically closely related SIVmon, SIVmus-1 and SIVgsn are not sensitive to cpz-TRIM5α, any of the three might have been involved in the recombination that gave rise to SIVcpz. Moreover, since chimpanzees are known to predate on a variety of lower primate species, the fact that cpz-TRIM5α does not seem capable of inhibiting SIV infection suggests that chimpanzees might acquire other SIV in the context of predation, which in turn may generate SIV with new abilities to adapt and spread into humans.

Hu-TRIM5α can block gammaretroviruses such as N-tropic murine leukemia virus (N-MLV), as well as lentiviruses such as feline immunodeficiency virus (FIV) and equine infectious anemia virus (EIAV) [24,78,79], but the sole known example of a primate retrovirus that is susceptible to hu-TRIM5α is ptERV1, an endogenous gammaretrovirus identified in the chimpanzee genome and absent in humans [80]. On the other hand, rh and agm-TRIM5α display a broad antiretroviral activity towards gammaretroviruses as well as lentiviruses, including primate lentiviruses [17,81]. Thus, the positive selective pressure on hu-TRIM5α is likely to be a retrovirus with a capsid conformation that is incompatible with primate lentivirus restriction. However, the ability of rh and agm-TRIM5α to restrict a wide range of lentiviruses reinforces the fact that these primate species have most likely been challenged by lentiviral infections.

The interaction of retroviral CA with CypA is a characteristic that was present in ancient retroviruses and that has been preserved throughout lentiviral evolution [82]. However, among “modern“ lentiviruses, the CypA-CA interaction is a widespread yet not universal feature [65,67]. While host CypA is essential for efficient HIV-1 replication in human cells, its role in controlling the infectivity of other lentiviruses remains unestablished [65]. The most critical target amino acids in CA for CypA binding are the glycine residue at position 89 (G89) and the proline residue at position 90 (P90) [83] although sequences flanking these two residues can also contribute significantly to CypA recognition [84]. Furthermore, Lin and Emerman have shown that the length of the loop that harbors G89 can also influence CypA interaction [65]. Our results with SIVgsn CA, which bound CypA domain of owl and mafa-TrimCyp, and CA of the related SIVmon and SIVmus-1, which did not, are consistent with the observations by others [85] that an A at position G89 maintains CypA binding while a Q does not. Interestingly, all SIVmon and SIVmus circulating isolates identified so far harbor a Q, while the SIVgsn isolates have an A89 [2,5]. The phylogenetic relationship between SIVmon, SIVmus-1, SIVmus-2 and SIVgsn could reflect ancient transmissions of an ancestor SIV to different primate species that shared the same habitats [2,77]. However, within this SIV lineage, only SIVgsn appears to bind owl-CypA, suggesting that SIVgsn CA may require the CypA interaction in its host species, while CA of SIVmon and SIVmus would have evolved without this requirement upon adaptation to their new hosts. Whether the CypA-CA interaction might be detrimental for the spreading of SIV into certain hosts will be more easily assessed after evaluating the interaction of the CA with the CypA of the natural host. As recently suggested, cyclophilins may have thus played a major role in the determination of SIVmac tropism [68].

CypA as well as Nup153 and Nup358/RanBP2 have recently been described to be a part of a pathway that mediates HIV-1 nuclear import [53-55,57] and CA which plays a major role in this property, determines the requirements for these proteins [45,52,54,59,63]. Since, we found that HIV-2_Rod_ CA exploits the same pathway, it would be of importance to test whether this dependence is shared by primary isolates of HIV in their natural host cells.

Nonetheless, we observed that Nup358/RanBP2 and Nup153 are not required for efficient infection by circulating SIV isolates and that the direct binding of Nup358/RanBP2 via its Cyp-like domain does not seem necessary. Interestingly, in HEK-293T cells, the knockdown of Nup358/RanBP2 or Nup153 has opposite effects on infectivity driven by some circulating SIV: we found that infectivity of viruses encoding CA derived from SIVmnd1 decreases with the former and increases with the latter. However, we demonstrated that the susceptibility to Nup358/RanBP2 depletion is not completely correlated to the CA-CypRanBP2 interaction. Indeed, only HIV-1 and HIV-2 were restricted upon infection of CHO cells stably expressing TRIM-RanBP2Cyp whereas SIVgsn and SIVmnd-1 were not (Figure 6C). As previously described we found that SIVmac was susceptible to Nup153 depletion [45,54] and in contrast to previous findings, was also susceptible to Nup358/RanBP2 depletion [63]. These results may be explained by a different protein knockdown approach (siRNA versus shRNA) and/or the different human cell lines used in the two studies (HEK-293T versus HeLa). Nevertheless, the proposed role of CypA in directing the virus towards the use of a nuclear entry pathway involving Nup358/RanBP2 and Nup153, as it has been suggested for HIV-1, does not seem to hold true with SIVmac, given its inability to bind CypA.

It will be interesting to test whether these circulating SIV isolates are susceptible to depletion of TNPO3, a karyopherin known to transport SR family proteins, which has been also implicated in the Nup358/RanBP2-Nup153 pathway [63]. Collectively, these results suggest that a pathway including at least Nup153, Nup358/RanBP2 and CypA for nuclear entry, is not conserved between primate lentiviral lineages. Therefore, it could also be interesting to study the relationship between Nup358/RanBP2-Nup153 in their respective host species during SIV infections.

## Conclusions

Productive infection of SIV in a new species involves necessary interactions with multiple cellular proteins within the infected host cell. Overall, we found that circulating SIV CA present a variety of phenotypes with regard to CA-interacting restricting or facilitating factors that do not appear to restrict these circulating SIV. Thereby, our observations highlight the use of distinct strategies to enter the nucleus, which may reflect species–specific requirements of co-factors by viruses. Understanding the lentiviral capsid/host protein interactions during the early steps of infection will be useful to determine the factors that contribute to a successful or dead-end simian-to-human transmission and also to further develop novel targets to hinder HIV replication.

## Methods

### Chimeric *gag*-*pol* SIVmac expression plasmids

The HIV-1 and HIV-1G89V gag fragments (matrix-capsid (MACA) or capsid (CA) were PCR amplified from the p8.91 and p8.91G89V *gag*-*pol* expression plasmids, respectively [25,86]. The HIV-2 MACA fragment was amplified from a HIV-2_ROD_ plasmid. MACA and CA SIV from the SIVgsn lineage were PCR amplified directly from uncultured lymphocyte DNA of *Cercopithecus* monkeys (*C*. *mona*, *C*. *cephus*, and *C*. *nictitans*) [5,77] and from the SIVcolCGU1 from mantled guereza (*Colobus guereza*) [87]. MACA and CA from SIVmnd1 were derived from PBMC DNA (a kind gift of Dr Roques, CEA, Fontenay-aux-Roses, France) obtained from an infected mandrill (*Mandrillus sphinx*) with SIVmnd1 GB1 strain [88]). PCR products were sub-cloned into the BssHII-ApaI (MACA) or the DraIII-AflII (CA) sites of a pUC19 plasmid containing an NdeI-SbfI fragment of a *gag*-*pol* SIVmac251 expression plasmid (pAd-SIV4) [89]. The pUC19/NdeI-SbfI vector was first subjected to *in vitro* mutagenesis with the QuikChange mutagenesis kit (Stratagene) to create BssHII and ApaI restriction sites by synonymous mutations. The chimeric NdeI-SbfI fragments were then cloned into an NdeI-SbfI digested pAd-SIV4 vector to obtain the chimeric *gag*-*pol* SIVmac expression plasmids.

### Generation of cells stably expressing TRIM5 variants

The full-length cDNA from different TRIM5α proteins (hu-TRIM5α, cpz-TRIM5α and agm-TRIM5α) were obtained from RT-PCR amplification of RNA from HeLa cells, from a chimpanzee (*Pan troglodytes verus*) PBMC (kindly provided by Dr. Heinz Ellerbrok, Robert Koch Institute, Berlin, Germany) and from Vero cells, respectively. The full-length cDNA from rh-TRIM5α and owl-TRIMcypA were PCR amplified from plasmids pLPCX-TRIM5αrh and pMIG-TRIMCyp, respectively, both were obtained from the NIH AIDS reagent program. Mafa-TRIMcypA and mamu-TRIMcypA were kindly provided by Dr. Greg Towers (University College London, UK) [90,91]. To construct TRIM-RanBP2CypA fusion protein, the RanBP2-Cyp like sequence was PCR-amplified from Jurkat cell cDNA and cloned in place of the CypA motif of owl-TRIMcypA. After purification, all amplified PCR products were subcloned into a pCR4-TOPO vector according to the manufacturer's instructions (Invitrogen), followed by sequencing of three independent clones. These inserts were then cloned into the NotI and BglII sites of a retroviral vector containing a C-terminal hemagglutinin (HA) derived from pLXSN [92]. To produce MLV particles, HEK-293T cells were then co-transfected with the pLXSN-based retroviral vectors encoding the various HA-tagged TRIM5 variants along with plasmids expressing MLV Gag-Pol (pC57GP) [93] and the vesicular stomatitis virus (VSV) G envelope glycoprotein (pCSIG) [94]. After 48 h, retroviral virions were harvested in supernatants and used to transduce CHO cells followed by selection in 200 μg/ml G418 (Invivogen).

### Infection with lentiviral virions harboring wt and chimeric capsids

HEK-293T and CHO cells were cultivated in Dulbecco modified Eagle medium (DMEM) supplemented with 10% fetal bovine serum, non-essential amino acids and 100 U penicillin/ml and 0.1 mg streptomycin/ml at 37°C and 5% CO2-air atmosphere.

HIV-1, SIVmac and chimeric SIV virions carrying the green fluorescent protein (GFP)-reporter gene were generated by co-transfecting HEK-293T cells with three expression vectors: (i) a *gag*-*pol* expression vector (p8.91 for HIV and pAd-SIV4 for SIVmac and chimeric SIV) (ii) VSV-G envelope glycoprotein expression vector (pCSI-G) allowing an efficient entry into the mammalian cell lines and (iii) GFP lentiviral viral vectors derived either from HIV (pSIN-CSGW) [95] or SIV (GAE-SFFV-GFP-WPRE) [89]. Virion-containing culture supernatant was harvested 48h after transfection and filtered through a 0.45 μm-pore-size filter. When necessary, retroviral supernatants were concentrated 100 fold by ultra-centrifugation through a 20% sucrose cushion in a SW28 rotor at 25000 rpm at 4°C for 2 hours. All viruses were stored at −80°C until use.

Viral stocks were titrated on non-restricting CHO cells by counting the number of GFP positive cells, by flow cytometry, two days after transduction. All infections were performed on 2×10^4^ cells in 96-well plates, with challenging viruses at M.O.I. that yielded 5 to 30% GFP-positive infected cells, in the presence of polybrene at a concentration of 4 μg/ml. When required, cyclosporin-A (CsA) (Sigma-Aldrich) was added during infection at a concentration of 5 μg/ml. Cells were then incubated at 37°C/5%CO_2_. GFP-positive cells were enumerated 48h later by flow cytometry on a FACSCalibur instrument (Becton Dickinson) and 10,000 events were collected. Data analyses were performed using FlowJo 7.6.1 software (Tree Star).

### siRNA transfection

Sequences of siRNA were as follows: 5′ - GGCAGCUCUACCAAAUGUtt - 3′ for the Nup153 (position 2593–2615) (NM_005124) [96]; 5′- GGCUCCAAAGAGCGGAUUUtt-3′ from Saitoh *et al*. [97], 5′-GCGCGAAAUUGUUUCGU-Utt-3′ (position 3719 to 3738) and 5′-GCA-AAC-CAC-GUU-AUU-ACU-Att-3′ (position 8704 to 9723) for Nup358/RanBP2 (accession number NM_006267.4).

HEK-293T cells were transfected with 300 pmoles of each specific RNA duplex using Oligofectamine (Invitrogen) according to the manufacturer’s instructions. A siRNA directed against firefly luciferase was used as control. Forty-eight hours after transfection, cells were trypsinized, plated in 96-well plates, infected with GFP reporter viruses and analyzed by flow cytometry 2 days later. In some experiments, a combination of multiple siRNAs was used (total of 300 pmol).

### Immunoblotting

CHO cells stably expressing HA-tagged TRIM5 variants were lysed in 100 mM NaCl, 50 mM Tris (pH 7.5) and 1% Triton X-100 containing protease inhibitors (Sigma-Aldrich). Proteins were separated on a 10% acrylamide gel, transferred onto PVDF membrane and probed with a rat anti-HA antibody (3F10, Roche Applied Science). A peroxidase-conjugated goat anti-rat (SouthernBiotech) was used as secondary antibody. Loading was controlled by probing with a β-actin antibody. For CA detection, conditioned cell-free supernatants of transfected HEK-293T cells were pelleted through a 20% sucrose layer in TEN Buffer (10 mM Tris.HCL pH7,5; 100 mM NaCL; 1 mM EDTA) at 25000 rpm for 2h30 at 4°C in a SW 40 Ti rotor. CA of pelleted viruses were monitored using sera from HIV-2 infected patients and anti-human-IgG-HRP IgG (Sigma-Aldrich) as primary and secondary antibodies, respectively.

Protein depletion by siRNA in HEK-293T cells was monitored by immunoblotting using a rabbit polyclonal antibody against Nup358/RanBP2 (ab64276, Abcam) and mouse Nup153 antibodies (ab24700 (Abcam) with a peroxidase-conjugated goat anti-rabbit and anti-mouse as secondary antibodies, respectively (Sigma-Aldrich). Lamin-B was used as a loading control.

### Computational structure predictions

The computer modeling of N-terminal CA structure from the SIVgsn lineage, SIVmnd1 and SIVcol was performed with the raptorX server http://raptorx.uchicago.edu) [70], using the experimental CA crystal structure of HIV-1 bound to human CypA (PDB: 1M9C; http://www.rcsb.org/pdb/explore.do?structureId=1m9c) as a structural template. The multiple sequence alignments of the target and template sequences were performed using ClustalX [98]. The predicted CA structures were visualized using the PyMOL 1.4.1 Linux version (http://pymol.org/citing).

## Competing interests

The authors declare that they have no competing interest.

## Authors’ contributions

Conceived and designed the experiments: JIM, MS, JLB, VC. Performed the experiments: JIM, VC. Analyzed the data: JIM, JLB, MS, VC. Wrote the manuscript: JIM, MS, JLB, VC. All authors read and approved the final manuscript.
